# Time-Action Analysis (TAA) of the Surgical Technique Implanting the Collum Femoris Preserving (CFP) Hip Arthroplasty. TAASTIC trial Identifying pitfalls during the learning curve of surgeons participating in a subsequent randomized controlled trial (An observational study)

**DOI:** 10.1186/1471-2474-9-93

**Published:** 2008-06-24

**Authors:** Jakob van Oldenrijk, Matthias U Schafroth, Mohit Bhandari, Wouter C Runne, Rudolf W Poolman

**Affiliations:** 1 Department of Orthopaedic Surgery, Academic Medical Centre, Amsterdam, The Netherlands; 2 Department of Orthopaedic Surgery, Onze Lieve Vrouwe Gasthuis, Amsterdam, The Netherlands; 3 Department of Orthopaedic Surgery, McMaster University, Hamilton, Canada

## Abstract

**Background:**

Two types of methods are used to assess learning curves: outcome assessment and process assessment. Outcome measures are usually dichotomous rare events like complication rates and survival or require an extensive follow-up and are therefore often inadequate to monitor individual learning curves. Time-action analysis (TAA) is a tool to objectively determine the level of efficiency of individual steps of a surgical procedure.

**Methods/Design:**

We are currently using TAA to determine the number of cases needed for surgeons to reach proficiency with a new innovative hip implant prior to initiating a multicentre RCT. By analysing the unedited video recordings of the first 20 procedures of each surgeon the number and duration of the actions needed for a surgeon to achieve his goal and the efficiency of these actions is measured. We constructed a taxonomy or list of actions which together describe the complete surgical procedure. In the taxonomy we categorised the procedure in 5 different Goal Oriented Phases (GOP):

1. the incision phase

2. the femoral phase

3. the acetabulum phase

4. the stem phase

5. the closure pase

Each GOP was subdivided in Goal Oriented Actions (GOA) and each GOA is subdivided in Separate Actions (SA) thereby defining all the necessary actions to complete the procedure. We grouped the SAs into GOAs since it would not be feasible to measure each SA. Using the video recordings, the duration of each GOA was recorded as well as the amount of delay. Delay consists of repetitions, waiting and additional actions. The nett GOA time is the total GOA time – delay and is a representation of the level of difficulty of each procedure. Efficiency is the percentage of nett GOA time during each procedure.

**Discussion:**

This allows the construction of individual learning curves, assessment of the final skill level for each surgeon and comparison of different surgeons prior to participation in an RCT. We believe an objective and comparable assessment of skill level by process assessment can improve the value of a surgical RCT in situations where a learning curve is expected.

## Background

### Introducing new techniques

Surgical expertise is a relatively under-explored item in surgical trials. Often, results of surgical trials are reported without information on the participating surgeons expertise in a new treatment option [1]. Critics used this argument against the utilization of randomized controlled trials (RCT) in surgery [2]. When surgeons plan an RCT comparing a well established implant with a relatively low morbidity to a new, potential superior, implant; a surgeon has to weigh the additional benefits to possible morbidity the new implant may give. Using innovative implants possibly exposes a patient to risks due to the surgeon's inexperience introducing the new surgical technique, perhaps leading to an increase of complications or decreasing the functional result.

Through clinical trials, functional result as well as complications can be evaluated. Clinical trials however evaluate the outcome of the procedure as a whole and are therefore inadequate to determine which specific pitfalls are inherent in the operation itself. By determining these pitfalls within the operation, the surgeon can possibly anticipate them and thereby avoid them.

Moreover, random variation of surgical outcome is influenced both by number of cases included and the frequency with which the outcome occurs. In circumstances where the expected outcome is rare, outcome indicators will have only limited power to detect real differences in quality [3]. A surgical technique with a low complication rate requires a high amount of surgical procedures with a long term follow-up for these complications to occur. This is particularly the case when evaluating the outcome of innovative prosthetic implants [4-6]. However, when introducing a new innovative implant it is important to identify pitfalls and difficulties at an early stage, preferably during the first few cases, thereby minimising risk for subsequent patients in a planned RCT evaluating the new implant. Identifying pitfalls of a surgical technique during a learning curve therefore ideally requires a system which:

- is capable of analysing individual steps of a surgical procedure

- requires a small amount of procedures (small number of cases)

- does not require a long follow-up and provides immediate feedback

- is able to detect these pitfalls

- is reproducible

This calls for process instead of outcome measurement. Outcome measures are most relevant for the broader perspective since they reflect the inter-play of a wide variety of factors. With a narrow perspective, on individual learning curves, process measurements become relatively more useful. Process measurements are often only considered useful if they are assumed to correlate with clinical outcome [3]. However, considering that errors within a process can have a synergistic effect it is important to focus on all errors not just those directly associated with a poor outcome [7]. Errors can be categorised by active and latent errors. Active errors have an immediate effect on outcome, while latent errors are hidden within the system, until their synergistic effects will accumulate with resulting adverse events [8,9].

Process measurement can be performed by two methods: Implicit and explicit. Implicit methods require expert judgement, without predefined criteria. Explicit methods assess quality of care against predefined criteria or algorithms, thereby providing standardised comparative and reproducible data [7]. In this study we aim to compare several surgeons and several procedures, necessitating an explicit reproducible measurement method. A previous pilot study performed at the Orthopaedic Research Centre Amsterdam by Veth and De Beer has shown an inter-observer reliability (expressed as IntraClass Correlation Coefficient) of 0.78 to 0.91 for anterolateral minimally invasive total hip arthroplasty, demonstrating it to be a reproducible tool to evaluate a perioperative process objectively.

### Time-action analysis

Time-action analysis (TAA) is a tool to objectively determine the level of efficiency of individual steps of a surgical procedure [10-13]. By analysing unedited video recordings of a surgical procedure the number and duration of the actions needed for a surgeon to achieve his goal and the efficiency of these actions is measured. In the past TAA has successfully been used to evaluate laparoscopic procedures and total shoulder arthroplasty procedures [10-13]. Utilizing TAA a surgeon is able to recognise and anticipate specific pitfalls. It can also be used as a tool to evaluate the improvement of efficiency and the decrease of errors in time as a representation of the learning curve. By comparing the surgical technique of experienced surgeons with inexperienced surgeons, difficult steps which need further emphasis during the learning curve can be identified and errors possibly avoided. Furthermore it can provide feedback to manufacturers of surgical materials, consequently they can determine how improvements in surgical material can increase efficiency. Even though conducting a fully efficient procedure does not ensure good clinical outcome, improvements in surgical techniques is the most important way in which a surgeon can alter the outcome.

### Femoral neck preserving hip arthroplasty

The Biodynamic cementless total hip prosthesis, introduced by Pipino in 1979, was designed with the aim to preserve maximum amount of bone stock for future revisions, to achieve physiological compression, tension and torsion forces and to minimise damage to vascular structures in the proximal femoral region [14,15]. The Biodynamic stem was replaced by the modified CFP prosthesis in 1996. The CFP prosthesis is made of titanium alloy with a hydroxyapatite porous coating, with longitudinal ribs to promote osseointegration. A seven year follow-up study of 353 CFP stem showed 99% good intergration, with only 2 cases of radiographically proven aseptic loosening [16]. It has a built in anatomic anteversion and 117° and 126° CCD angles to closely resemble physiological anatomy. Clinical follow-up showed good functional recovery and DEXA analysis of 10 patients showed minimal periprosthetic bone loss [14,16,17,17]. The CFP stem is combined with a Trabeculae Oriented Pattern (TOP) cementless hemispheric cup. The TOP cup has a biequatorial dissociation with a medialcaudal recess to allow a wider range of motion and a cranial rim reaching over 180° to reduce the risk of dislocation [14]. A clinical follow-up study of 301 TOP cups showed no detachement, migration, or osteolysis after 7 years [16].

Currently the CFP stem has not been compared with conventional straight stems in an RCT, therefore additional benefits remain to be determined. Implantation of the CFP stem requires a major alteration of operating technique compared to conventional straight cementless stems and require specially designed instruments. Therefore a substantial learning curve is to be expected potentially compromising the validity of an RCT due to differential expertise based bias. Determining the minimum number of cases required before participating in an RCT is often arbitrary and for many complex procedures the learning curve has not reached a plateau after reaching the required number of procedures [2]. Another potential for differential expertise bias is the influence of the number of years of clinical experience of participating surgeons [2].

We aimed to evaluate the learning curve of experienced hip surgeons who will be participating in a future RCT evaluating the CFP stem. We hypothesized that using TAA could result in a clear understanding of a surgeon's learning curve and finally surgical expertise before embarking in an RCT comparing the CFP stem with a straight stem in total hip arthroplasty. By determining a surgeon's learning curve prior to initiating an RCT we aim to determine and possibly reduce the influence of differential expertise bias.

### Main objective/research question

What is the number of cases needed for surgeons with different levels of experience to reach proficiency with the CFP stem arthroplasty?

### Secondary objective/research question

Do surgeons with much experience learn a new technique more rapidly than a surgeon with less experience?

### Hypothesis

We hypothesize that after 10–15 cases, surgeons will reach a plateau of the level of efficiency. We expect to register a significant decrease of errors during this period. Furthermore we expect no correlation to exist between change of functional outcome or complication rate and case number.

### Study design

We propose a prospective observational study of 4 surgeons across 2 centres using Time-action analysis to analyse the level of efficiency of the surgical technique of surgeons with different levels of experience with the CFP stem and with varying clinical experience.

## Methods

### Outcome

#### Primary outcome parameters

• Nett Goal Oriented Action Time = time spend on Goal Oriented Actions (GOAs) – delay

• Delay = time spend on repetitions, additional actions and waiting

• Efficiency = percentage of nett Goal Oriented Action Time during each procedure

• Level of difficulty = Nett Goal Oriented Action Time of a specific GOA + time spend on repeating that GOA during subsequent GOAs

#### Secondary outcome parameters

##### Functional outome

As part of current clinical protocol, functional outcome will be measured using the Dutch version of the Hip disability and Osteoarthritis Outcome Score (HOOS), validated by De Groot et al [18]. Functional score will be recorded pre-operatively and at 6 weeks, 3 months and one year follow-up, as part of standard follow-up protocol [18]. Follow-up protocol is not affected by this study.

##### Position of the prosthesis

position of the prosthesis will be measured on AP-pelvis x-rays by two independend assessors blinded for the case number and level of experience.

##### Complication rate

complications: peri-operative and post-operative will be recorded by independent nurse practitioners blinded for the case number.

### Participating centres

The study will be a cooperation between the Academic Medical Centre Amsterdam, the Onze Lieve Vrouwe Gasthuis (OLVG) Amsterdam, The Isala Hospital Zwolle and McMaster University Hamilton, Canada. Surgical procedures will be performed by several surgeons at the OLVG Hospital in Amsterdam and the Isala Hospital in Zwolle. The learning of each individual surgeon will be analysed. Analysis will be performed at the Academic Medical Centre in Amsterdam by an independent assessment committee.

### Variables

#### Independent variable

Level of experience

We aim to compare 4 surgeons with different levels of experience in implanting non-cemented stems. Experience will be defined in years post board registration and number of arthroplasties performed per year.

#### Dependent variables

Four types of actions are analysed:

1. Goal Oriented Actions (GOA)

2. Repetitions

3. Additional Actions (AA)

4. waiting

#### Controlled variables

• Interval between operations

For valid comparison of the learning curve, time between procedures cannot exceed more than 1 month.

• Patient population

Inclusion criteria of patients

Adult patients with disabling degenerative arthritis of the hip or (avascular) head necrosis.

Maximum age is determined by the current clinical protocol of each participating hospital.

Exclusion criteria of patients

Patients not eligible for CFP arthroplasty in this study are:

Patients with a BMI of more than 40

Patients with skeletal immaturity

Patients with a life expectancy of less than 3 years

Patients with altered anatomy resulting in impossibility for the CFP procedure, according to the surgeon e.g.:

-hip dysplasia with high dislocation

-post traumatic severe anatomy change

Patients with extremity amputation

Patients with an active malignant disease or current cytostatic treatment

• Surgical technique

Predefined in the taxonomy

### Sample size

Since there is currently no data on the learning curve of the CFP stem, sample size is estimated by reviewing the learning curve of other implants or techniques in current literature. Previous literature on learning curves in total hip and total knee arthroplasty determine the learning curve on the amount of complications, clinical outcome, implant position or mean operative time [6,19].

Archibeck et al. analysed the first experience of 159 surgeons with the 2-incision minimally invasive THA. He found a significant reduction in the mean operative time in the first ten cases. The amount of complications did not show any reduction as a function of the case number [19].

Flamme et al. studied the learning curve in total hip arthroplasty of three surgeons with different levels of experience operating on a total of 168 patients. They found no association between functional outcome as determined by the Merle d'Aubigné score and case number. However considering intraoperative complication rate, all three surgeons ended their learning curve after a maximum of 20 cases [6]. Based on these studies, we estimate the efficiency to reach a plateau between 10 and 20 cases. Therefore the first 20 cases of each surgeon will be analysed.

A learning curve can however differ between individual surgeons. It is therefore important to study multiple surgeons with different levels of experience and to define a stopping rule. The stopping rule defines whether the estimated number of 20 cases is sufficient to study an individuals learning curve and terminate the task analysis or whether additional cases are needed. The criterion for terminating the task analysis was previously determined through the probability of failure (P) multiplied by the cost of failure (C) to an acceptable level – the P × C rule [20]. The role of the P × C rule is to save the analyst time in analysing tasks where the error variance would be inconsequential and to guide more exploration where the error variance would be intolerable [20]. The cost of failure is inherent to the clinical consequence of a peri-operative complication which requires clinical interpretation. We defined three major peri-operative complications during total hip arthroplasty which determined C:

1. massive bleeding

2. fracture (femoral or acetabular)

3. evident neurological damage

This resulted in the following definition of the stopping rule: if a surgeon encounters a major complication (C) within the last five consecutive surgical procedures (P), the stopping rule is considered unacceptable and an additional five surgical procedures have to be analysed.

To facilitate comparison of different levels of expertise we suggest one surgeon less than five years board certification, two surgeons 5 to 15 years and 1 surgeons more than 15 years board certification. This justifies our total sample of 4 surgeons each performing 20 cases, resulting in a total of between 80 surgical interventions to be analysed.

### Data acquisition

With the use of three cameras and a microphone, video-recordings are made of abovementioned procedures. One wide-angle (or fish eye) camera is positioned proximally 1,5 meters above the operating table, to record an overview-image of the operating theatre. A second camera is situated on the head of the surgeon to record a clear view of the actions performed by the surgeon. An additional portable camcorder is used to film any actions that are missed by the previously mentioned cameras. Sound is recorded with use of the camcorder. The total number of persons required to be present in the operating room during the operation is limited to only one.

**Figure 1:**
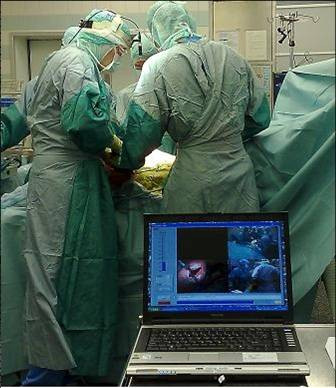
Setup in the Operating Theater.

A Quad unit combines four signals, 3 video and 1 sound, into one signal. This way, all images can be analysed simultaneously on one laptop on a split-screen (see Figure 1 and see Additional file 1: movie sample TAA).

### Equipment

Theatre setup

• 3 camera's

◦ One camcorder camera on a stand

◦ One camera to be placed on the head of the surgeon

◦ One fish eye camera to be placed above the operating theatre

• Quad unit

• AV-converter

• Software for video analysis and image conversion

• Laptop with digital video portal.

### The Taxonomy

Time-action analysis is a quantitative method which can be used to objectively analyse the course of a surgical procedure. The analysis is done by scoring strictly defined actions in time. This way a procedure can be observed in detail and knowledge can be obtained about the difficulty and efficiency of a procedure and the comparison of two procedures can be performed objectively.

In performing a time-action analysis a taxonomy is used; a list of predefined Goal-Oriented Actions (GOA) which; taken together, describe the total procedure. The concept of time-action analysis described by Minekus [12] served as a template for the design of this taxonomy and has been modified for the CFP stem surgical technique. A GOA is defined as an action which contributes directly to the progress of the procedure [12]. The GOA's can then be divided in a number of Separate Actions (SA). For example, in the GOA *skin incision*, the SA's are: skin incision and positioning retractors. By formulating the GOA's as accurate as possible, almost every recorded action can be defined. This way of grouping separate actions into GOA's was chosen, since it would not have been feasible or efficient to separately score every action seen in a 90 minute film. Eventually the operative procedure is divided in Goal Oriented Phases (GOP);

1- Incision phase,

2- Prosthesis phase, subdivided in

a. femoral phase

b. acetabulum phase

c. stem phase

3- Closure phase.

These GOP's are divided in GOA's and at last, the GOA's are subdivided in SA's. All essential actions of the procedure are defined, e.g. sawing, cutting, suction, coagulating, placing of retractors. Extra sections are added for 'waiting' and 'additional actions' to be able to eventually account for every second of film.

Apart from formulating this list of pre-defined steps it is very important to come to good general agreements on the methods of scoring so that the inter-observer variation is limited to a minimum (see Table 1 in Additional file 2).

### Construction of the taxonomy

Using a method similar to Sarker et al. a template taxonomy is constructed using currently available literature on CFP stem surgical techniques [21,21]. The template taxonomy is discussed with participating surgeons and adjusted to represent individual surgical technique. This results in the final taxonomy, which is defined prior to analysis. During the course of the analysis the structure of the taxonomy is fixed.

### Task analysis

After scoring the video, a task analysis will be performed using the predefined taxonomy. This results in an hierarchical (studying procedural sequences) task analysis.

**Figure 2:**
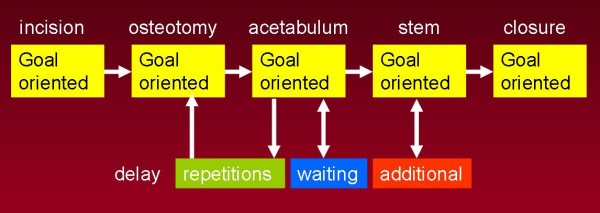
**Graphic representation of the entire surgical procedure:** 5 Goal Oriented Phases (GOP) and possibilities for delay. Repetitions, Waiting and Additional Actions.

The duration and number of all actions are categorised in (Figure 2):

1. Goal Oriented Actions (GOA's)

2. Repetitions

3. Additional actions (AA's)

4. Waiting

GOA's are actions which contribute directly to the advancement of the procedure. Furthermore they are performed in consecutive order during each fase corresponding with the pre-defined taxonomy.

A GOA inadequately executed may require a Repetitions. Repetitions are Separate Actions (SA's), which are repeated due to insufficient progress of the procedure. An example of a Repetition is inadequate exposure, which causes insufficient progress and necessitates extending the incision, or repositioning the retractors.

A surgeon may encounter difficulties requiring actions not defined in the taxonomy. These actions are defined as AA's. This may be either due to abnormal anatomy, unexpected pathology, instrument failure or an inadequately executed goal-oriented action. Since we merely describe the External Mode of Malfunction, instead of the mechanisms or causes of malfunction we do not subcategorise additional actions by their cause. As Rassmussen put it: "For performance in unfamiliar situations requiring proper problem solving, a cognitive task analysis, if at all possible, requires interviews and discussions with the performer [22]." This results in a subjective analysis of an opinion. To obtain objective data additional actions will be categorised irrespective of their cause.

Waiting is the amount of time between two actions, with a minimum of 5 seconds.

While the amount and duration of GOA's is an expression of the level progress during the operation, delay is defined by the amount of time spend on Reptitions, AA's and waiting. The level of efficiency is an expression of the level of progress and delay.

### Outcome measurements

If the outcome is unacceptable, despite having a perfectly efficient procedure, the procedure has still failed to reach its goal. In task analysis it is therefore important to have an objective method to verify that the goal of the procedure has been met. Goal of the operation is determined by functional outcome as perceived by the patient and position of the prosthesis.

#### Functional outcome

To measure postoperative functional outcome the validated Dutch version of the Hip disability and Osteoarthritis Outcome Score (HOOS) taken pre-operative, within one week before the operation and postoperative at 6 weeks and one year follow-up [18]. Questionnaires will be collected by local surgical staff and send to the trial committee.

#### Position of the prosthesis

AP pelvis roentgenograms are taken pre-operatively, postoperatively and during follow-up visits after 6 weeks and one year follow-up as part of the standard care.

##### Cup inclination

The radiological cup inclination as described by Murray [23] defined as the angle between the longitudinal axis and the acetabular axis when this is projected on to the coronal plane. It is measured by measuring the angle between the teardrop line and the line which divides the cup inner ellipse in halve. If the teardrop line is not clearly visible, alternatively the line between the two SI joint or the lower border of the ramus inferior can be used.

Femoral shaft alignment is measured on AP pelvis roentgenogram.

##### Leg length discrepancy

pre- and postoperative leg length discrepancy is determined on a standing weight-bearing AP pelvis roentgenogram after calibration. A horizontal line is drawn through the inferior aspect of the acetabular teardrop. Two other lines parallel to the teardrop line are drawn through the centre of the lesser trochanter of each femur. Leg length discrepancy is defined as the difference between distances from the teardrop line to the lines through the lesser trochanters of each femur. All radiographs will be analyzed by two independent surgeons, blinded to the case sequence and surgeon's expertise.

#### Complications

Postoperative complications during the follow-up will be recorded by an independent nurse practitioner.

##### Assessor

Task analysis will be performed by an independent observer.

##### Data analysis

A regression analysis will be performed to reveal any significant increase of efficiency or decrease of the errors rate as a function of the case number.

### Patient safety

#### Interventions

The TAASTIC trial is strictly an observational study, therefore the study must not interfere with clinical decision making. Allocation of the CFP stem to a patient is not influenced by this trial. No additional interventions will be performed to facilitate this trial.

#### Privacy

Video recordings will only be made after written informed consent by the patient. The identity of the patients is concealed in all recordings.

#### Ethical approval

Since the TAASTIC trial is an observational study, a formal ethical approval was waived for this study by the OLVG Medical Ethics Committee. The OLVG Medical Ethics Committee declared to have no objections to the TAASTIC trial.

#### Operating theatre protocol

Cameras must not compromise sterility in the operating theatre. The minimum additional person to be present during surgery is restricted to only one. Local theatre protocols apply.

#### Trial management

### Organisation

#### Coordination

Responsible for coordination of the study is the Surgical Learning Curve and Expertise committee consisting of the following members:

Jakob van Oldenrijk, Academic Medical Centre, Amsterdam

Matthias Schafroth, Academic Medical Centre, Amsterdam

Rudolf Poolman, Onze Lieve Vrouwe Gasthuis (Principal Investigator), Amsterdam

Mohit Bhandari, McMaster University, Hamilton, Canada

#### Operating surgeons

During the course of the CFP TAASTIC trial The Surgical Learning curve and Expertise Working Group consist of the following surgeons:

R.W. Poolman, Onze Lieve Vrouwe Gasthuis, Amsterdam

W.C. Runne, Onze Lieve Vrouwe Gasthuis. Amsterdam

C.C.P.M. Verheyen, Isala Kliniek Zwolle

C. van Egmond, Isala Kliniek Zwolle

## Competing interests

The TAASTIC trial will receive financial contribution for an amount of less than €10.000,- from Link Nederland B.V. Prior to receiving any contribution an Unrestricted Grant Agreement or financial disclosure will be signed by Link Nederland B.V. and the trial committee members. Link shall financially contribute to the study, but will not be considered a Sponsor of the study. Principal Investigator (RWP) shall be responsible for the general management and supervision of the study. Link Nederland B.V. is not involved with the study design; in the collection, analysis, and interpretation of data; in the writing of the manuscript; and in the decision to submit the manuscript for publication.

## Authors' contributions

JVO designed the protocol, will carry out the data acquisition, data analysis and data interpretation and wrote and edited the final manuscript. MUS was involved with the design of the protocol, data acquisition, trial consultant and edited the final manuscript. MB was involved with protocol design, was an important data analysis consultant and edited the final manuscript. WCR was involved with the protocol design, will perform the surgical interventions and edited final manuscript. RWP designed the protocol, is principal investigator, will perform the surgical interventions, edited the final manuscript. All authors read and approved the final manuscript.

## Pre-publication history

The pre-publication history for this paper can be accessed here:



## Supplementary Material

Additional file 1 Movie sample of a video recording during the stem phase. Click here for file

Additional file 2 Table 1: Scoring agreements. Click here for file
