# Supplementary material for: Time-Action Analysis (TAA) of the Surgical Technique Implanting the Collum Femoris Preserving (CFP) Hip Arthroplasty. TAASTIC trial Identifying pitfalls during the learning curve of surgeons participating in a subsequent randomized controlled trial (An observational study)
Source: BMC Musculoskelet Disord. 2008 Jun 24;9:93. doi: 10.1186/1471-2474-9-93 (PMC2483707; doi:10.1186/1471-2474-9-93)
Supplement: Additional file 2 — Table 1: Scoring agreements. [file 1471-2474-9-93-S2.doc]

| **Action** | **definition** | **scoring method** |
| --- | --- | --- |
| Goal Oriented Phase **(GOP)** | The total procedure is divided in 5 Goal Oriented Phases: Incision / femural / acetabulum / stem / closure. The total duration of a group of Goal Oriented Phases = the gross Goal Oriented Phase time | A GOP ends when the first GOA of the subsequent GOP is initiated |
| Goal Oriented Actions **(GOA)** | All Goal Oriented Phases are subdivided in Goal Oriented Actions. The total duration of a group of Separate Actions = gross Goal Oriented Action time | A GOA ends when the first SA of the subsequent GOA is initiated. |
| Separate Actions **(SA)** | Each individual action is scored as a Separate Action. A group of Separate Actions is a Goal Oriented Action. | A SA is put "**on hold**" when the subsequent SA within the same GOA is initiated. If a SA is repeated within one GOA, the separate action is NOT scored as a repetition, but the duration is added to the total duration of the initial SA. (see definition of repetitions). An SA ends when the first SA of the subsequent GOA is initiated. The duration of SA's within one GOA are not recorded separately. Only the total duration of each GOA is recorded. The duration of SA's are recorded when they are considered to be either Repetitions or AA's. |
| Repetitions | The duration of a Separate Action belonging to an already finished Goal Oriented Action, initiated during a subsequent Goal Oriented Action. | If an SA of a previous GOA is executed during a subsequent GOA the duration of this SA is separately scored as a repetition. |
| Waiting | Any action or the absence thereof, resulting in a temporary discontinuation of the surgical procedure (with a minimum duration of 5 seconds) | examples: waiting for instruments, self-assembly of instruments, explanation or discussion. Ends with the initiation of the subsequent SA. |
| Additional Actions **(AA)** | Additional Actions (AA) are SA's not predefined in the taxonomy, and essential for the continuation of the surgical procedure. | Every AA measured during a surgical procedure for the first time is recorded in a separate AA list. The same term will be used for similar subsequent AA's |
